# Modulation of the Physical Properties of 3D Spheroids Derived from Human Scleral Stroma Fibroblasts (HSSFs) with Different Axial Lengths Obtained from Surgical Patients

**DOI:** 10.3390/cimb43030121

**Published:** 2021-10-22

**Authors:** Hiroyasu Katayama, Masato Furuhashi, Araya Umetsu, Fumihito Hikage, Megumi Watanabe, Hiroshi Ohguro, Yosuke Ida

**Affiliations:** 1 Departments of Ophthalmology, Sapporo Medical University School of Medicine, Hokkaido 060-8556, Japan; h.katayama@sapmed.ac.jp (H.K.); araya.alaya.favreweissth@gmail.com (A.U.); fuhika@gmail.com (F.H.); watanabe@sapmed.ac.jp (M.W.); ooguro@sapmed.ac.jp (H.O.); 2 Cardiovascular, Renal and Metabolic Medicine, Sapporo Medical University School of Medicine, Hokkaido 060-8556, Japan; mfuruhas@gmail.com

**Keywords:** 3D spheroid culture, myopia, axial length, human scleral stroma fibroblasts (HSSFs)

## Abstract

In the current study, to elucidate the pathological characteristics of myopic scleral stroma, three-dimensional (3D) cultures of human scleral stroma fibroblasts (HSSFs) with several axial lengths (AL, 22.80–30.63 mm) that were obtained from patients (*n* = 7) were examined. Among the three groups of ALs, <25 mm (*n* = 2), 25–30 mm (*n* = 2), and >30 mm (*n* = 3), the physical properties of the 3D HSSFs spheroids with respect to size and stiffness, the expressions of extracellular matrix (ECM) molecules, including collagen (COL) 1, 4, and 6 and fibronectin (FN) by qPCR and immunohistochemistry (IHC), and the mRNA expression of ECM metabolism modulators including hypoxia-inducible factor 1A (HIF 1A), HIF 2A, lysyl oxidase (LOX), tissue inhibitor of metalloproteinase (TIMP) 1–4, and matrix metalloproteinase (MMP) 2, 9, and 14 as well as several endoplasmic reticulum (ER) stress-related factors were compared. In the largest AL group (>30 mm), the 3D HSSFs spheroids were (1) significantly down-sized and less stiff compared to the other groups, and (2) significant changes were detected in the expression of some ECMs (qPCR; the up-regulation of *COL1* and *COL4*, and the down-regulation of *FN*, IHC; the up-regulation of COL1 and FN, and down-regulation of COL4). The mRNA expressions of ECM modulators and ER stress-related genes were also altered with increasing AL length (up-regulation of *HIF2A*, *MMP2, XBP1,* and *MMP14*, down-regulation of *LOX, TIMP 2* and *3*, *GRP78*, *GRP94*, *IRE1,* and *ATF6*). In addition, a substantial down-regulation of some ER stress-related genes (*ATF4*, *sXPB1* and *CHOP*) was observed in the 25–30 mm AL group. The findings presented herein suggest that small and stiffer 3D HSSFs spheroids in the largest AL group may accurately replicate the pathological significance of scleral thinning and weakening in myopic eyes. In addition, the modulation of several related factors among the different AL groups may also provide significant insights into our understanding of the molecular mechanisms responsible for causing myopic changes in the sclera.

## 1. Introduction

The sclera, the major outer layer of the eye ball, serves to protect the interior ocular structures as well as functioning as a determinant of their shape and size. Structurally, the sclera is largely composed of collagens (COLs) with interspersed fibroblasts, in which several extracellular matrix molecules (ECMs) are produced and maintained [1]. It is also well known that the sclera is the most important determinant of myopia pathogenesis. In fact, during the development and progression of myopia, several changes occur, including scleral thinning and weakening, and those changes further induce pathological complications in the retina and choroid that can include maculopathies, retinal schisis, and detachment, especially in highly myopic eyes [2,3,4]. 

A possible mechanism of the myopia-associated thinning of the sclera leading to axial length (AL) elongation includes the simultaneous acceleration in scleral ECM degradation and the slow production of new ECM molecules [3,5]. It has been suggested that the activation of matrix metalloproteinases (MMPs) and decreased activity of tissue inhibitor of metalloproteinases (TIMPs) are included in this process [6,7]. In fact, the reduced production of new ECM molecules, particularly COL1, and the diminished production of proteoglycans [8] as well as morphological observations, such as COL fibers with smaller diameters and fewer COL fiber bundles, can be recognized within high myopic eyes [9]. As of this writing, although the underlying mechanism responsible for causing such myopia-associated changes remains unidentified, it is speculated that these complex changes may be caused by (1) differences in the nature of the human scleral stroma fibroblasts (HSSFs) between myopic and non-myopic eyes, (2) currently unknown factors other than HSSFs, such as several growth factors, cytokines or others, or both (1) and (2). However, to answer this question, a suitable in vivo cell culture model that replicates the myopic changes of three-dimensional (3D) human scleral architecture will be required. 

Therefore, in the current study, to develop such an in vivo model for replicating the human scleral architecture, HSSFs that were obtained from seven patients with several ALs were used to produce 3D drop cell cultures, leading to the successful production of 3D spheroids from various human fibroblasts [10,11,12,13]. The obtained 3D HSSFs spheroids were categorized into three different AL groups based on length, (1) <25 mm, (2) 25–30 mm, and (3) >30 mm, and the following aspects were compared these among groups: their physical properties, size and stiffness, ECM expression by qPCR and immunohistochemistry, and gene expressions of ECM modulator, hypoxia-inducible factor 1A (HIF 1A), HIF 2A, lysyl oxidase (LOX), tissue inhibitor of metalloproteinase (TIMP), and matrix metalloproteinase (MMP) as well as several endoplasmic reticulum (ER) stress-related genes. 

## 2. Materials and Methods

The current study, which was conducted at the Sapporo Medical University Hospital, Japan, was approved by the institutional review board (IRB registration number 282-8) and according to the tenets of the Declaration of Helsinki and national laws for the protection of personal data. Informed consent was obtained from all subjects who participated in this study. 

### 2.1. Preparation of 3D Spheroid Cultures of Human Scleral Stroma Fibroblasts (HSSFs)

Specimens of scleral tissue were obtained from 7 surgical patients who had been treated by a scleral shortening procedure (the demographic data for these 7 patients are shown in Supplementary Table S1) and further processed for the isolation and preparation of the human corneal stroma fibroblasts (HSSFs) according to a previously described method [14] with minor modifications. Then, the HSSFs cells were grown in 150 mm 2D culture dishes until reaching 90% confluence at 37 °C in 2D growth medium composed of HG-DMEM containing 10% FBS, 1% L-glutamine, and 1% antibiotic-antimycotic and were maintained by changing the medium every other day. These 2D cultured HSSFs that were prepared were further processed for 3D spheroids culture using a hanging droplet 3D spheroid culture plate (# HDP1385, Sigma-Aldrich) as described in our previous report [10]. Briefly, 2D cultured HSSFs, as described above, were washed with phosphate buffered saline (PBS), and the cells were detached by treatment with 0.25% trypsin/EDTA. After centrifugation for 5 min at 300× *g*, the cell pellet was re-suspended in 3D sphenoid medium composed of the 2D growth medium supplemented with 0.25% methylcellulose (Methocel A4M) as a stabilizer of the morphology of the 3D spheroids. Approximately 20,000 HSSFs in the 28 μL of the 3D spheroid medium were subjected into each well of the hanging drop culture plate (Day 0), and thereafter, every following day, half of the medium was replaced until Day 5.

### 2.2. Measurement of 3D HSSFs Spheroid Sizes

Bright-field phase contrast microscopy images of 3D spheroids were obtained in a ×4 objective lens using an inverted microscope (Nikon ECLIPSE TS2; Tokyo, Japan). The largest cross-sectional area (CSA) of the 3D HSSFs spheroid was measured using the Image-J software version 1.51 n (National Institutes of Health, Bethesda, MD, USA).

### 2.3. Immunohistochemistry of 3D HSSFs Spheroids

Immunohistochemistry of the 3D HSSFs spheroids was performed by previously described methods with minor modifications [11,15]. All procedures were performed at room temperature unless otherwise stated. Briefly, 3D HSSFs spheroids prepared, as described above, were fixed in 4% paraformaldehyde in PBS overnight, blocked in 3% bovine serum albumin (BSA) in PBS for 3 h, and washed twice with PBS for 30 min. Then, they were incubated with an anti-human COL1, COL4, COL6, or FN rabbit antibody (1:200 dilutions) at 4 °C overnight, washing 3 times with PBS for 1 h each, incubation with 1:1000 dilutions of a goat anti-rabbit IgG (488 nm), phalloidin (594 nm), and DAPI for 3 h, and mounting with ProLong Gold Antifade Mountant with a cover glass. A series of the axial immunofluorescent images with a 2.2 μm interval during 35 μm from their surface were obtained by a Nikon A1 confocal microscope using a ×20 air objective with a resolution of 1024 × 1024 pixels. Regarding the signal intensity of the 3D HSSFs spheroids, the maximum intensity/surface area was measured at 35 μm from the top of the spheroid in the z-plane. The surface area was calculated as follows: surface area = D × A/(A + π × H2), where D (μm) indicates organoid diameter, A (μm2) indicates area of sectioned spheroid, and H (μm) indicates height (= 35 μm).

### 2.4. Quantitative PCR and Solidity Measurement of 3D Spheroids

Quantitative PCR following total RNA extraction, reverse transcription using specific primers as shown in Supplementary Table S2, and the physical solidity measurements using a micro-squeezer of 3D spheroid were performed as described in a previous study [16].

### 2.5. Statistical Analysis

Using Graph Pad Prism 8 (GraphPad Software, San Diego, CA, USA), comparison of two mean values by a two-tailed Student’s t-test and the difference analysis among groups by two-way analysis of variance (ANOVA) followed by Tukey’s multiple comparison test were performed. All data are expressed as the arithmetic mean ± standard error of the mean (SEM).

## 3. Results

**Figure 1:**
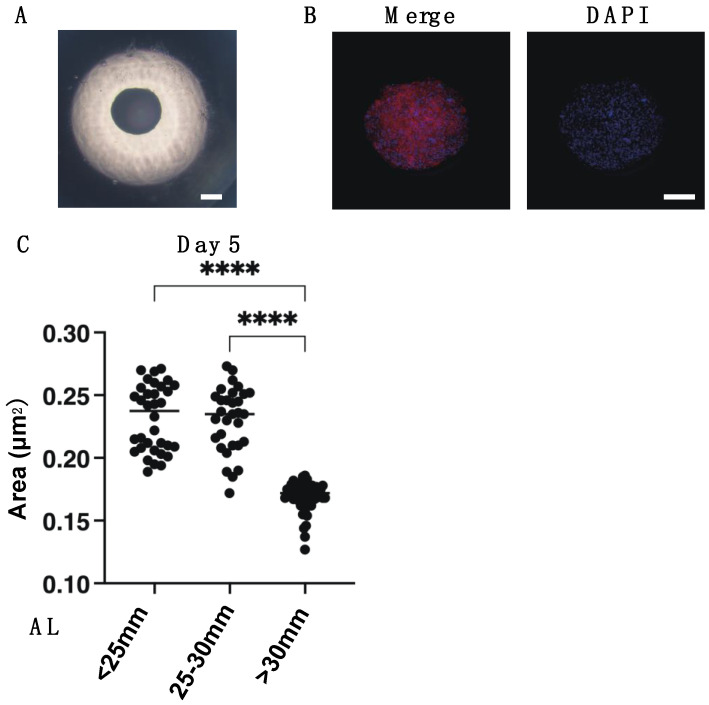
Representative images by phase contrast microscopy (**A**) and immunohistochemistry (**B**), and the mean sizes of the 3D spheroids of human scleral stroma fibroblasts (HSSFs) with various axial lengths (ALs) (**C**). Representative images by phase contrast (PC, panel A) and immunolabeling with DAPI and phalloidin (scale bar: 100 µm) (panel B) of 3D HSSFs spheroids at Day 5 are shown. Among three different ALs, <25 mm, 25–30 mm, or >30 mm, the mean sizes of the 3D HSSFs spheroids are plotted in panel C. All experiments were performed in duplicate using fresh preparations (*n* = 15 or 5 for size measurement or PC and immunolabeling images, respectively, in each experimental group). Data are presented as the arithmetic mean ± standard error of the mean (SEM). **** *p* < 0.001 (ANOVA followed by Tukey’s multiple comparison test were examined).

**Figure 2:**
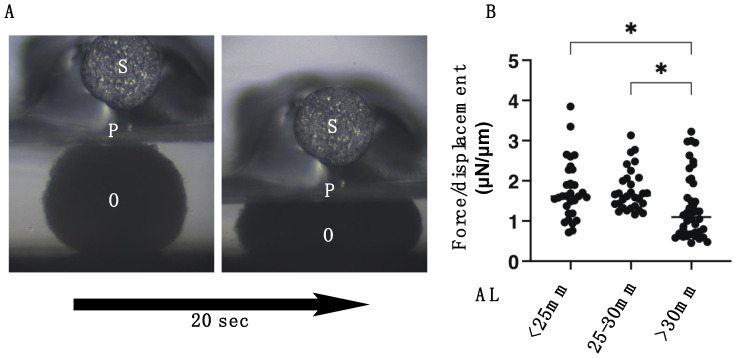
Physical solidity of the 3D spheroids of human scleral stroma fibroblasts (HSSFs). For the measuring the physical stiffness of the 3D HSSF spheroids, a micro-squeezer analysis was used, as shown in **panel A** (O: 3D sphenoid, P: compressing plate, S: pressure sensor). Among three different ALs, <25 mm, 25–30 mm, or >30 mm, each living 3D HSSFs sphenoid at Day 5 (*n* = 20–30) was compressed to induce a 50% deformity during a period of 20 s. The force required (μN) to accomplish this was measured, and the resulting force/displacement (μN/μm) values were plotted in **panel B**. Data are presented as the arithmetic mean ± standard error of the mean (SEM). * *p* < 0.05 (ANOVA followed by Tukey’s multiple comparison test).

To elucidate the pathological involvement of human scleral stromal fibroblasts (HSSFs) within the myopia etiology, 3D spheroid cultures of HSSFs obtained from seven patients with several axial lengths (ALs, 22.80–30.63 mm) were employed. Consistent with our previous studies using human orbital fibroblasts (HOFs) [11,12] and human conjunctival fibroblasts (HconFs) [17], uniform, round-shaped 3D spheroids of HSSFs were successfully produced during the 5-day culture (Figure 1A). Nuclear staining with DAPI indicated (1) no artifacts or cell death, and (2) multiple layers of concentrically aligned HSSFs cells were observed within the interiors of the 3D HSSFs spheroids (Figure 1B). Since these histological observations of our prepared 3D HSSFs spheroids replicated the morphology of human scleral stroma reasonably well [18], this system was used in the present study. We categorized these 3D HSSFs spheroids into three different groups based on length: <25 mm (*n* = 2), 25–30 mm (*n* = 2), and >30 mm (*n* = 3), and their physical properties, mean sizes, and stiffness were compared. Quite interestingly, the 3D HSSFs spheroids of the largest AL group (>30 mm) were significantly smaller (Figure 1C) and less stiff (Figure 2) than the corresponding values for the other groups. This result clearly indicated that HSSFs obtained from longer ALs could form 3D spheroids, thus reproducing myopia-associated changes, scleral thinning, and weakness. 

**Figure 3:**
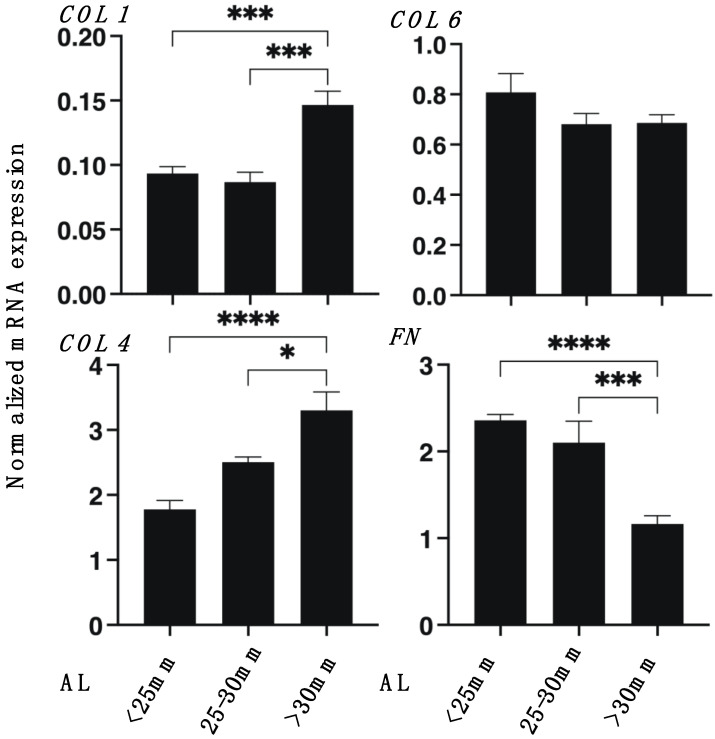
qPCR analysis of ECM in the 3D spheroids of human scleral stroma fibroblasts (HSSFs). Among three different ALs, <25 mm, 25–30 mm, or >30 mm, 3D spheroids of HSSFs at Day 5 were subjected to a qPCR analysis of ECM proteins including *COL1*, *COL4, COL6,* and *FN*. All experiments were performed in duplicate using fresh preparations (*n* = 5, each). Data are presented as the arithmetic mean ± standard error of the mean (SEM). * *p* < 0.05, *** *p* < 0.005, **** *p* < 0.001 (ANOVA followed by Tukey’s multiple comparison test).

**Figure 4:**
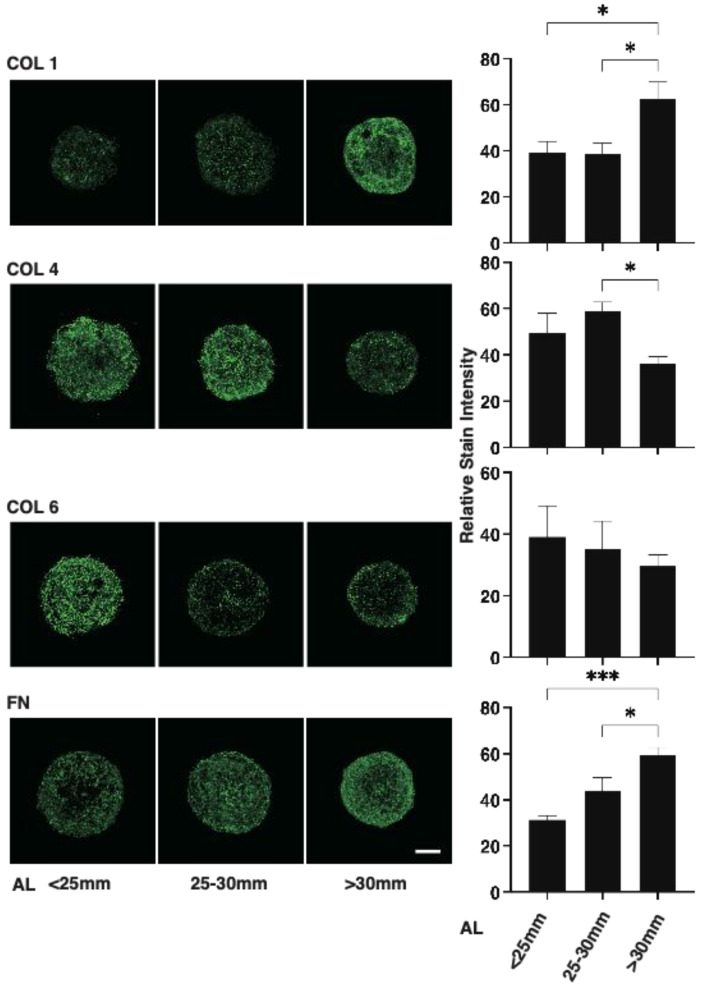
Immunolabeling of ECM in the 3D spheroids of human scleral stroma fibroblasts (HSSFs). The three different ALs <25 mm, 25–30 mm, or >30 mm of 3D spheroids of HSSFs were subjected to immunolabeling of ECM proteins including COL1, COL4, COL6, and FN at Day 5. Representative images and plots of the relative staining intensities for each ECM are shown in panels A and B, respectively. All experiments were performed in duplicate using fresh preparations (*n* = 5, each). Data are presented as the arithmetic mean ± standard error of the mean (SEM). * *p* < 0.05, *** *p* < 0.005 (ANOVA followed by Tukey’s multiple comparison test).

To further characterize the 3D HSSF’s spheroids among the three different AL groups, the expressions of major ECM proteins of HSSFs, COL 1, 4, and 6 and FN were analyzed by qPCR and immunohistochemistry. As shown in Figure 3, the mRNA expression of *COL1* and *4* or *FN* of the largest AL group (>30 mm) were significantly up-regulated and down-regulated, respectively, as compared with the other groups. However, in contrast to this, immunostaining indicated a significant increase in COL1, a substantial decrease in COL4, and an increase in FN in the largest AL group (>30 mm) as compared to other groups (Figure 4). Since immunohistochemistry adequately reflects the spatial expressions of target molecules within the 3D spheroid, the levels of gene expressions and immunolabeling are frequently not consistent. In fact, such diversity in levels between gene expressions and immunolabeling was also recognized in our previous studies using 3D spheroids of HOFs [11] or human trabecular meshwork (HTM) [13]. 

**Figure 5:**
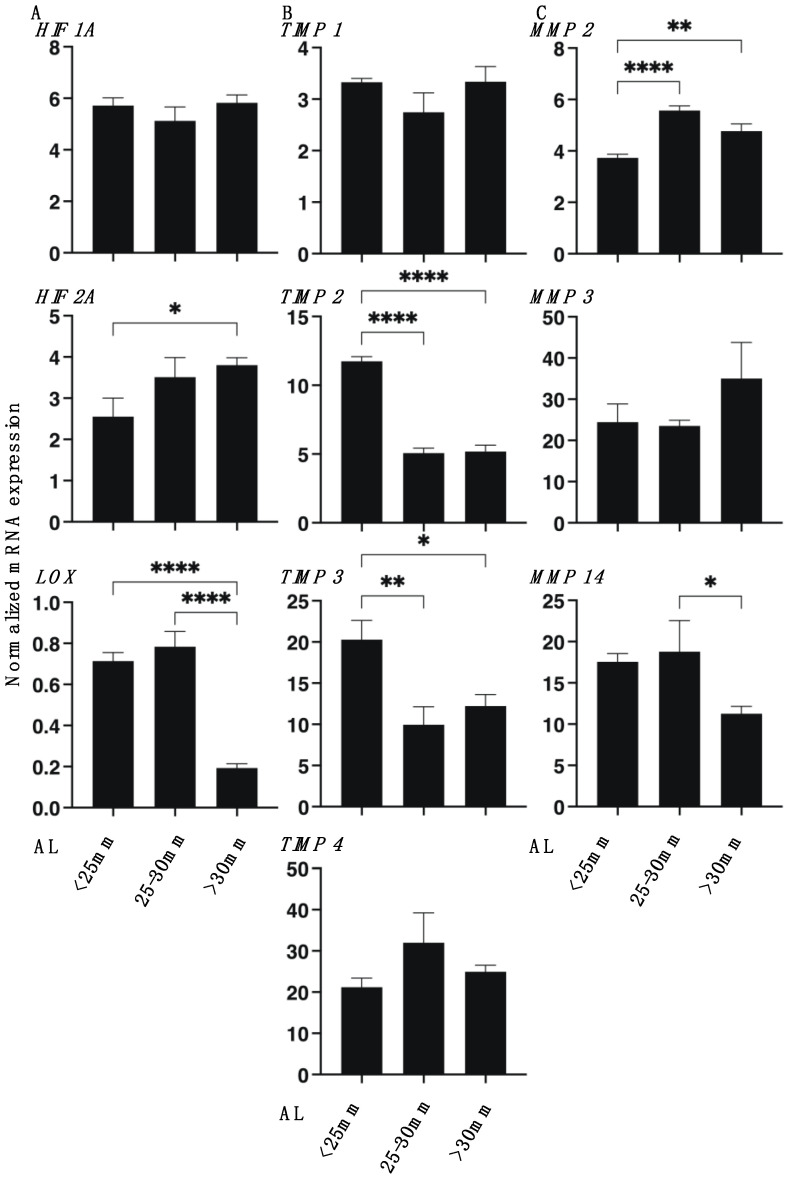
mRNA expression of HIF 1A, HIF 2A, LOX (**A**), TIMPs (1–4) (**B**), and MMPs (2, 9, 14) (**C**) in 3D spheroids derived from human scleral stroma fibroblasts (HSSFs). The three different ALs <25 mm, 25–30 mm, or >30 mm of 3D spheroids of HSSFs were subjected to qPCR analysis at Day 5 and the mRNA expression of *HIF 1A*, *HIF 2A*, *LOX* (**panel A**), *TIMP 1–4* (**panel B**), and *MMP 2, 3, 9,* and *14* (**panel C**) was estimated. No evidence for the expression of MMP9 was found. All experiments were performed in duplicate using fresh preparations (*n* = 5, each). Data are presented as the arithmetic mean ± standard error of the mean (SEM). * *p* < 0.05, ** *p* < 0.01, **** *p* < 0.001 (ANOVA followed by Tukey’s multiple comparison test).

**Figure 6:**
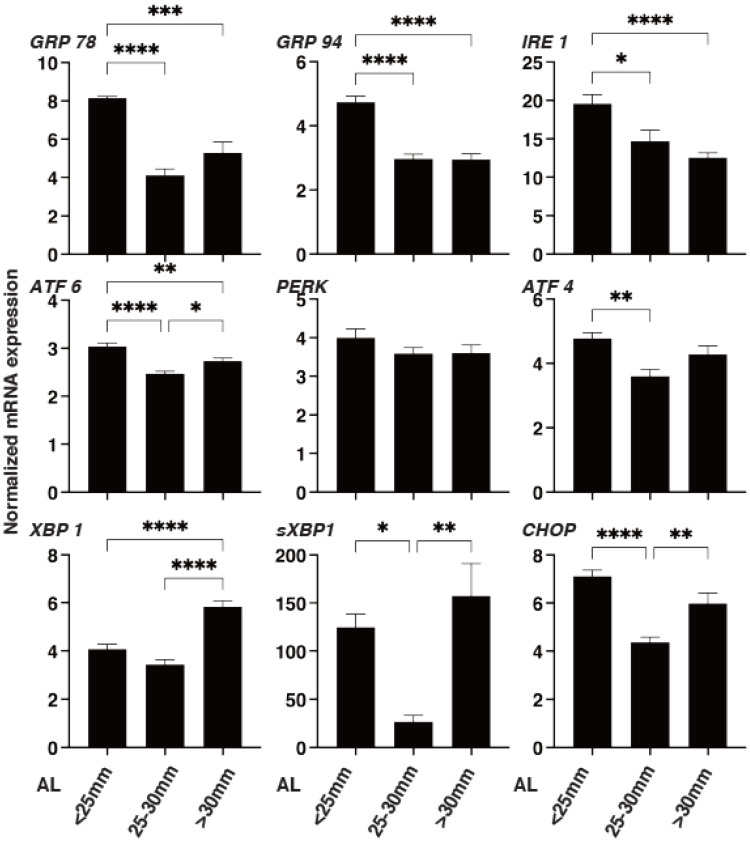
mRNA expression of major ER stress-related genes in the 3D spheroids of human scleral stroma fibroblasts (HSSFs). The three different ALs, <25 mm, 25–30 mm, or >30 mm, of 3D spheroids of HSSFs were subjected to qPCR analysis at Day 5 to estimate the mRNA expression of major ER stress-related factors including protein kinase RNA-like endoplasmic reticulum kinase (PERK), activating transcription factor 6 (ATF6), and inositol-requiring enzyme 1 (IRE1), and their downstream factors including the glucose regulator protein (GRP)78, GRP94, the X-box binding protein-1 (XBP1), spliced XBP1 (sXBP1), and CCAAT/enhancer-binding protein homologous protein (CHOP). All experiments were performed in duplicate using fresh preparations. Data are presented as the arithmetic mean ± standard error of the mean (SEM). * *p* < 0.05, ** *p* < 0.01, *** *p* < 0.005, **** *p* < 0.001 (ANOVA followed by Tukey’s multiple comparison test).

To examine this issue further, we studied the mRNA expression of ECM modulators, HIFs, LOX, TIMPs (1–4), and MMPs (2, 9, and 14) as well as ER stress-related genes of three master regulators: protein kinase RNA-like endoplasmic reticulum kinase (PERK), activating transcription factor 6 (ATF6), and the inositol-requiring enzyme 1 (IRE1), as well as their downstream factors, including the glucose regulator protein (GRP)78, GRP94, the X-box binding protein-1 (XBP1), spliced XBP1 (sXBP1), and CCAAT/enhancer-binding protein homologous protein (CHOP). As shown in Figure 5, *LOX*, *TIMP 2* and *3,* and *MMP14* were substantially down-regulated, and *HIF 2A* and *MMP 2* were substantially up-regulated, respectively, with increasing AL length. Similarly, as shown in Figure 6, *GRP78*, *GRP94*, *IRE1,* and *ATF6* were down-regulated, and *XBP1* was up-regulated in the larger AL groups. These collective findings indicate that modulation of the gene expressions of regulators of ECM metabolism, such as *HIF 2A, LOX*, *MMPs,* and *TIMPs*, and several ER stress factors may be involved in the pathogenesis of myopia. However, and interestingly, a significant down-regulation of *ATF4*, *sXBP1,* and *CHOP* was observed in the case of the AL 25–30 mm group, but not in the AL > 30 mm group, which suggested that a currently unknown etiology may be at play between the mild (AL 25–30 mm) versus the severe myopic changes (AL > 30 mm groups).

## 4. Discussion

The human sclera is the fibrous connective outer layer of the eye ball is largely composed of COL fibers and fibroblasts. Human scleral stroma fibroblasts (HSSFs) produce and secrete ECM components during the development of the eye [2,5,19,20]. When the developing eye reaches adult size at approximately 10 years of age, ECM synthesis is decelerated [21], and thereafter, physiological scleral ECM remodeling continues throughout the lifetime of an individual [22]. During the course of the metabolism of ECM components in the human sclera, a currently unknown myopia pathogenesis could lead to scleral thinning and weakness, which is particularly evident in the posterior segment as well being recognized as posterior staphyloma [23]. Histopathological studies have demonstrated that scleral tissue remodeling may be an important process based upon the following facts: (1) in myopic sclera, COL fibrils become smaller in diameter and abnormally arranged [23], and (2) fibroblast proliferation is decelerated and down-regulated ECM proteins in human and primate eyes [24,25]. Such anatomical and histopathological studies on the human myopic eye are only available via the use of postmortem ocular tissues. Furthermore, several animal models have also been extensively used in attempts to elucidate the mechanism underlying the pathogenesis of myopia and to develop potential therapies [26,27,28,29,30,31,32,33]. However, a valid and suitable in vivo human myopia model has not been developed yet, and this has prevented a better understanding to be developed regarding the molecular pathogenesis of myopia, especially in terms of myopia-associated scleral thinning and weakness. In the current study, 3D HSSFs spheroids were successfully prepared using surgically obtained scleral specimens from seven patients with different ALs. Among the three different AL groups (<25 mm, 25–30 mm, and > 30 mm), 3D HSSFs spheroids of the largest AL groups were significantly smaller and less stiff as compared with the others, suggesting that 3D HSSFs spheroids replicated the phenotype of myopic scleral manifestations reasonably well, and thus, this may be an applicable in vivo pathogenic model for studies related to myopia. 

MMPs are proteases that degrade various ECM components including collagen, fibronectin, and other molecules, and TIMPs function as regulatory inhibitors of these processes [34]. Among the MMP isoforms, it has been consistently reported that elevated MMP2 activity is associated with myopia in animal studies, although less is known regarding their inhibitory regulator TIMP2 and related molecules such as TGF-β [2,6]. In fact, the up-regulation of TIMP1 and TIMP2 were reported in a myopic model of tree shrews [35], but a decreased or unaltered TIMP2 expression were reported in another study [2]. Therefore, it appears that elevated MMP2 activity is important and is likely involved in myopia pathogenesis, since MMP2 has collagenase and gelatinase activity, which could then lead to scleral degradation. In fact, upon myopia pathogenesis, the deceleration of the fibroblast proliferation and the down-regulation of COL1 and glycosaminoglycans were reported in both human and primate eyes [24,25]. In the current study, a significant up-regulation of MMP2 and down-regulation of TIMP2 and 3, and MMP14 were also recognized in the 3D HSSFs spheroids of the larger AL groups, as was also observed in previous studies [2,6] as above. While in contrast, our study identified different changes in the mRNA expression of ECM molecules, that is, the up-regulation of *COL1* and *4*, and the down-regulation of *FN*. Although we currently do not specifically know why the expression of COL1 was different between the current study and previous studies [24,25], we speculate that the pathogenic phase may be different; that is, our 3D spheroid cultures reflected an earlier phase of myopic changes, while previous studies [24,25] may have reflected a later steady state of myopic changes. To support our speculation, differences in the changes of ECM expression (up-regulation of COL1 and FN and down-regulation of COL4) by immunohistochemical analysis and substantial fluctuations in the expression of ER stress-related genes suggested that active changes had progressed by the time that the matured 3D HSSFs spheroids had formed. 

Hypoxia-inducible factor-1 (HIF-1) and HIF-2 belong to the HIF family of basic helix–loop–helix Per–Arnt–Sim transcription factors. The activation of HIF-1 and HIF-2 mediates physiological adaptations to sustained hypoxia [36] and regulates cellular metabolism and ECM remodeling in hypoxic pathophysiological conditions [37]. Among the downstream factors of HIFs, lysyl oxidase (LOX), an extracellular and copper-dependent monoamine oxidase that catalyzes the cross-linkage of lysine residues within COLs, can result in HIF-dependent tissue fibrosis [38,39]. As of this writing, only HIF 1A has been reported to be involved in the pathogenesis of myopia [40,41,42,43], and thus, this represents the first demonstration of the ALs-related alteration of the HIF 2A and LOX signaling. 

Concerning the limitations of this current investigation, the numbers of patient specimens used were relatively small (*n* = 7). Since significant biological variabilities exist from patient to patient, a study using only a few HSSFs cell sources may be insufficient for determining whether the obtained effects are representative across all patient tissues/cells. In addition, the mechanism responsible for causing the ECM remodeling associated with myopia pathogenesis has not yet been elucidated. However, there were several interesting clues regarding elucidating these mechanisms. For example, the current analysis indicated an opposite trend for MMP14 in comparison to MMP2 and MMP3. Therefore, further studies with the objective of identifying the currently unknown upstream regulatory mechanisms responsible for causing such ECM remodeling by blocking several possible factors by specific inhibitors and Si RNA using larger numbers of HSSFs obtained from patients will be required.

## Data Availability

All data are shown in the article.

## Supplementary Materials

The following are available online at https://www.mdpi.com/article/10.3390/cimb43030121/s1, Table S1. The demographic data of the patients. RRD: rhegmatogenous retinal detachment, MHRD: macular hole retinal detachment. Table S2. Sequences of primers and Taqman probes used.
